# Unveiling *Crocosphaera* Responses to Phosphorus Depletion: Insights From Genome Analysis and Functional Characterization

**DOI:** 10.1111/1462-2920.70153

**Published:** 2025-07-14

**Authors:** Chloé Caille, Sophie Rabouille, Eva Ortega‐Retuerta, Yann Denis, Olivier Crispi, Barbara Marie, Mireille Pujo‐Pay, Vladimir Daric, Emmanuel Talla, Amel Latifi

**Affiliations:** ^1^ Sorbonne Université, CNRS Laboratoire D'océanographie Microbienne, LOMIC Banyuls‐sur‐Mer France; ^2^ Aix Marseille Univ, CNRS Laboratoire de Chimie Bactérienne LCB, IMM Marseille France; ^3^ Plateforme Transcriptomique, Aix‐Marseille Univ., CNRS, IMM Marseille France; ^4^ Sorbonne Université, CNRS, Biologie Intégrative Des Organismes Marins (BIOM) Banyuls/Mer France

**Keywords:** *Crocosphaera*, cyanobacteria, diel cycle, gene expression, genomics, phosphorus depletion

## Abstract

Unicellular, nitrogen‐fixing cyanobacteria (UCYN) thrive and support primary production in oligotrophic oceans, playing a significant role in the marine nitrogen cycle. *Crocosphaera* sp., a model organism for studying marine nitrogen fixation, is adapted to low phosphate (P_i_) concentrations. Yet, how *Crocosphaera* copes with P_i_ depletion is rather poorly understood. We present a genomics analysis of P_i_ stress‐responsive genes in this genus, encompassing six *C. watsonii* and two strains isolated in coastal environments, *C. subtropica* and *C. chwakensis*. We identified genes involved in P_i_ signalling and uptake, and dissolved organic phosphorus (DOP) hydrolysis. Results showed different genetic potentials to cope with P_i_ scarcity between the *Crocosphaera* strains. Physiological monitoring of cultures of *C. watsonii* WH8501 exposed to P_i_ depletion highlighted a capacity to survive for at least nine days, albeit with a skewed C:N:P stoichiometry. Upon addition of DOP, cultures efficiently recovered to a growth rate and cell composition equivalent to those observed under favourable conditions. The concomitant transcription analysis revealed diel expression patterns of P_i_‐related genes and endogenous clock genes, suggesting a possible circadian regulation. Our data deepen our understanding of the growth strategies *Crocosphaera* employs in P_i_‐limited environments, offering broader insights into microbial resilience in marine ecosystems.

## Graphical abstract

Genomic analysis, diel gene expression profiling, and physiological monitoring were combined to uncover how the unicellular cyanobacteria *Crocosphaera* thrive in low‐phosphate environments. Our results indicate that the strategic use of dissolved organic phosphorus (DOP) is central to its adaptation, providing new insights into microbial resilience in nutrient‐limited oceans.

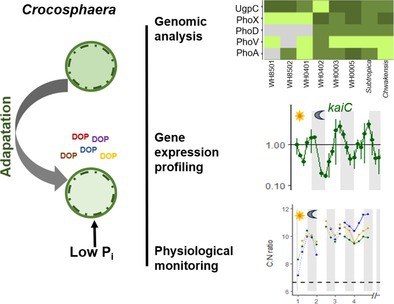

## 1 Introduction

Unicellular, nitrogen‐fixing cyanobacteria (UCYN) are prevalent in the tropics and subtropical areas of the open ocean, playing a significant role in the marine nitrogen cycle (Zehr and Capone 2020). Nitrogen (N) fixation is the primary mechanism introducing new N into the open ocean, supporting up to half of the new primary production in oligotrophic regions such as the subtropical North Pacific Ocean (Karl et al. 1997). This underscores the critical importance of diazotrophic cyanobacteria in oligotrophic regions. Extrapolating the growth yields of diazotrophs observed in the Northern Pacific to the global ocean, Montoya et al. (2004) estimated that nitrogen fixation may support up to 10% of the primary production at the global scale. The genus *Crocosphaera* is a cultivated representative of open‐ocean, photo‐autotrophic UCYN. It is a key model organism for studying N fixation and how this process varies with environmental factors. The *Crocosphaera* genus was divided into two clades based on the *nifH* gene distribution. The UCYN‐B clade includes *C. watsonii* (Zehr et al. 2001; Abed et al. 2009), subdivided into small and large strains (Bench et al. 2013). The UCYN‐C clade comprises *C. subtropica* and *C. chwakensis*, strains isolated in coastal environments and previously classified under *Cyanothece* (Mareš et al. 2019). The growth of diazotrophic cyanobacteria can be limited by the availability of nutrients, primarily iron and phosphorus (P). *Crocosphaera* strains are widely distributed in oligotrophic regions, where phosphate (P_i_) concentrations are very low (Zehr and Capone 2020). Their geographical distribution suggests that *Crocosphaera* strains have developed adaptive mechanisms to cope with P_i_ depletion.

Bacterial strategies to adapt to P_i_ limitation are diverse, including the replacement of phospholipids with sulfolipids, the use of high‐affinity P_i_ transporters (Rao and Torriani 1990), and the scavenging of dissolved organic phosphorus (DOP) sources through hydrolytic enzymes (Yamane and Maruo 1978; Wu et al. 2007; Abed et al. 2009). Most of these mechanisms are induced in response to P_i_ depletion, as perceived by the two‐component regulatory system of the PhoB‐PhoR family (Santos‐Beneit 2015). Current knowledge on how *Crocosphaera* copes with P_i_ depletion is primarily derived from studies on *C. watsonii* . For example, alkaline phosphatase activity (APA) was measured in *C. watsonii* P_i_‐depleted cultures, and this strain can use dissolved organic phosphorus (DOP) as its sole phosphorus source when provided as phosphomonoesters and phosphodiesters (Rabouille et al. 2022). However, despite this ability to utilize DOP through APA, there was no observed transcriptional induction of the genes considered to encode the AP enzyme when P_i_ was limited (Dyhrman and Haley 2006; Pereira et al. 2016). Thus, the genes responsible for encoding the inducible AP likely remain unidentified.

When extracellular P_i_ is abundant, *C. watsonii* employs the constitutively expressed phosphate inorganic transport (Pit) system to import phosphorus into the cell (Dyhrman and Haley 2006). Under P_i_ limiting conditions, a high‐affinity phosphate‐specific transport (Pst) system, which uses ATP‐mediated transport, is activated, and the gene encoding the high‐affinity phosphate‐binding protein (PstS) is upregulated to enhance P_i_ uptake (Pereira et al. 2016, 2019). Comparative genome analysis of six unicellular *C. watsonii* strains belonging to the two morphological phenotypes (small and large cells) revealed a variation in the copy number of genes involved in the transport and use of P_i_ (Bench et al. 2013). Whole‐genome transcription analysis of *C. watsonii* cultures showed that 47.4% of the genes exhibited a diel expression pattern (Shi et al. 2010). In P_i_‐depleted cultures, the *pstS* gene also displayed a diel expression pattern (Pereira et al. 2016), indicating that adaptive mechanisms to cope with P_i_ scarcity might follow diel regulation, likely controlled by the circadian KaiABC clock (Kondo and Ishiura 2000). Initially identified in the unicellular cyanobacterium *Synechococcus elongatus* PCC7942 (Ishiura et al. 1998), the circadian clock is largely conserved within cyanobacteria (Kondo and Ishiura 1999). But, to our knowledge, the circadian clock has not yet been studied in *Crocosphaera*.

The results summarized above point to results specific to *C. watsonii* , while a comprehensive comparative overview of the genetic potential of the genus *Crocosphaera* to cope with P_i_ limitation is lacking. Here, we present a dual approach to the genetic and physiological response to P_i_ limitation. First, to compare the genetic potential of the different *Crocosphaera* strains, we conducted a functional genomics analysis of P_i_ depletion‐responsive genes across eight *Crocosphaera* genomes, including the six known *C. watsonii* strains and the coastal strains *C. subtropica* and *C. chwakensis*. In each genome, we identified all genes involved in P_i_ uptake, the hydrolysis of dissolved organic phosphorus (DOP), and the perception and regulation of gene expression in response to P_i_ limitation. Second, we analysed the expression of these genes during P_i_ depletion in cultures of *C. watsonii* WH8501. To characterize the consequences of P_i_ starvation and DOP use on the metabolism, we also concomitantly monitored the physiological parameters of cells in conditions of P_i_ depletion and during a recovery phase in which DOP was provided to the P_i_‐depleted cultures.

## 2 Experimental Procedures

### 2.1 Bioinformatics Analysis

#### 2.1.1 Datasets

In September 2023, we downloaded genome data for eight *Crocosphaera* strains from the NCBI FTP site (ftp:/ftp.ncbi.nih.gov/genomes/), selecting only those with the highest assembly levels (complete genome, scaffold, or contig) and excluding metagenome‐derived assemblies (strains DT_26, ALOHA_ZT_9, and MO_202). Genome details, including RefSeq categories and annotations, are provided in Table S2 (Sheet 1). Cell‐size data for *C. watsonii* (small/large) were sourced from the literature (Bench et al. 2013). We obtained Hidden Markov Models (HMMs) for protein families (Pfam v35.0) and Pgap (v4.0) from ftp.ebi.ac.uk/pub/databases/Pfam/releases/ and ftp.ncbi.nlm.nih.gov/hmm/4.0/, respectively. Reference seed proteins (52 total, listed in Table S2, Sheet 2) were retrieved from UniProt, prioritizing cyanobacterial orthologs and using *E. coli* proteins as alternatives when unavailable.

#### 2.1.2 Functional Domain Search for Orthologs Identification

The HMMER package (Mistry et al. 2013) and HMM domain profiles were used to identify seed functional domains in reference proteins, along with their domain organization (Table S2, Sheet 2). Alignments scoring Pfam/Pgap above the trusted cutoffs (Mistry et al. 2013) yielded 76 reference seed domains (Table S2, Sheet 3). HMMER3 and custom Perl scripts then searched for orthologs in *Crocosphaera* genomes, requiring at least one seed domain per protein. For overlapping domains, the longest with the best E‐values were selected. Orthologs were further analysed to confirm their domain patterns, retaining only those with strict seed domain presence (Hmm‐homologues). When two or more seed proteins shared identical Pfam domain patterns, Pgap domains were used to distinguish between them. To enhance sensitivity, a Blast search (E‐value ≤ 10^−5^) was performed, with significant alignments defined as MinLrap ≥ 0.8 and MaxLrap ≥ 0.8 (Blast‐orthologs). The Hmm‐ortholog and Blast‐ortholog sets were merged into a final ortholog set for each reference protein.

Reference proteins with identical Pfam/Pgap domain patterns were grouped for comparative analysis. This was the case for GlpQ and UgpQ [Leading to GlpQ‐UgpQ]; PhnC and PtxA [PhnC‐PtxA]; PhnE and PstC [PhnE‐PstC]; PstS and SphX [PstS‐SphX]; and UgpA and UgpE [UgpA‐UgpE].

To assess key genes in phosphate (Pst), phosphite (Ptx), phosphonate (Phn), and glycerol (Ugp) systems, tBlastN was performed against genomic sequences (E‐value ≤ 10^−5^). The best hit for each seed protein (classified by localization: periplasmic, inner membrane, or cytoplasmic) was considered the most likely ortholog.

### 2.2 Strain and Growth Conditions

*Crocosphaera watsonii* strain WH8501, isolated in the western tropical South Atlantic Ocean, was grown under obligate diazotrophy in a YBCII culture medium (Chen et al. 1996), which we slightly modified to make sure the medium was devoid of any source of N (Fe‐NH_4_‐citrate was replaced with Fe‐citrate). Cultures were kept at 27°C under a 12:12 h dark: light cycle at 600 μmol photons.m^−2^.s^−1^ to ensure light‐saturated growth. The growth medium was prepared from aged Mediterranean Sea water, collected at the Microbial Observatory Laboratoire Arago (MOLA) station (at 19 miles off the coast, 42°27.200′ N—03°32.600′ E, 500 m depth, salinity 38). The MOLA sampling point is a typical, oligotrophic, P_i_‐depleted environment. Water from this location is thus particularly appropriate to cultivate organisms isolated in oligotrophic waters. The collected water was aged for at least 8 weeks in the dark at 20°C, filtered through 1 and 0.22 μm polycarbonate filters, and autoclaved before use. The weeks‐long aging of the water before filtering, autoclaving, and preparing the medium, ensure that the nutrients present at the time of sampling have been consumed. The remaining macro‐ and micronutrients, if any, are then negligible compared to those added to prepare the medium. Before the experiment, we analysed the bacterial contamination in the strain. Three strains of heterotrophic bacteria were identified: *Nitratireductor aquibiodomus* , *Qipengyuania goetbuli*, and *Oceaniradius stylonematis*. Bacterial contamination was then monitored during the experiments by flow cytometry with SYBR Green staining (see below). Considering a carbon content of 4 to 8.9 fmol C.cell^−1^ in bacterial cells (Gundersen et al. 2002), the bacterial contamination represented between 3.63% ± 1.23% and 7.7% ± 2.48% of the total biomass. The changes in the biomass C:N:P ratio predominantly reflect *Crocosphaera* composition since the medium lacks any nitrogen sources other than vitamins, indicating that the contaminating bacteria depend exclusively on *Crocosphaera* exudates for nutrition. Furthermore, the genetic analysis was specifically designed to detect only gene expressions from *Crocosphaera*.

### 2.3 Experimental Design

A phosphate‐replete culture of *C. watsonii* WH8501 (grown with 50 μmol L^−1^ KH₂PO₄) was exponentially grown (0.26 d^−1^, R^2^ = 0.95) as inoculum for all replicates. Once the biomass hit 10^7^ cells mL^−1^, the culture was filtered and distributed equally into six flasks with P_i_‐depleted medium, targeting an initial concentration of ~3.5 × 10^6^ cells mL^−1^. Due to volume constraints, each replicate consisted of two 2.5 L flasks (Figure S1).

The six cultures were maintained under P_i_ depletion for 9 days. All culture replicates were started simultaneously and monitored daily for cell abundance to verify that all population dynamics were equivalent. After five days (ensuring P_i_ starvation), high‐frequency monitoring began on the sixth day (designated Day 1 in figures). Sampling occurred every 3 h over four days: starting at the dark–light transition (L0), then L3, L6, L9, D0, D3, D6, D9 (L = light, D = dark). The first triplicate was sampled for three days; sampling then shifted to the second triplicate after the light–dark transition of Day 3 (D0) due to volume depletion (Figure S1).

After nine days of P_i_‐depletion, recovery was induced in the second triplicate. Cultures were diluted in 2X YBCII medium (P_i_‐free) with 50 μmol P L^−1^ dissolved organic phosphorus (DOP: equal parts adenosine diphosphate (C_10_H_15_N_5_O_10_P_2_, Sigma‐Aldricht, ≥ 95%), alpha glycerophosphate (C_3_H_7_MgO_6_P · xH_2_O, Sigma‐Aldricht, 85%), nitrophenyl phosphate ((O_2_NC_6_H_4_O)_2_P(O)OH, Sigma‐Aldricht, 99%), and glucose 6‐phosphate (C_6_H_11_Na_2_O_9_P · xH_2_O Sigma‐Aldricht, ≥ 98%), each at 12.5 μmol P L^−1^). For DOP addition, 1 L from each culture replicate was transferred to 750 mL of DOP‐enriched medium. After three days of recovery, high‐frequency monitoring resumed for two days (Figure S1). The final DOP concentration in the cultures after dilution was 21.4 μmol P L^−1^. Since the DOP compounds contained nitrogen, dilution also introduced DON at an estimated final concentration of ~24 μmol N L^−1^.

### 2.4 Analytical Methods

#### 2.4.1 Cell size and abundance

*C. watsonii* WH8501 cell abundance was measured using a Coulter Cytoflex flow cytometer (Beckman) via auto‐fluorescent detection (Gasol and del Giorgio 2000; Veldhuis and Kraay 2000; Marie et al. 2014) Samples were collected daily during the P‐replete phase and after inoculation in P_i_‐depleted medium, then every 3 h during high‐frequency monitoring. Samples were fixed with 0.5% glutaraldehyde and stored at‐80°C. Heterotrophic bacterial contamination was assessed by flow cytometry after SYBR Green staining. Two acquisition procedures distinguished *Crocosphaera* and bacteria based on size and fluorescence, using 10 μm Trucount beads (BD Biosciences) as a standard. Trucount bead solutions were analysed in triplicate to confirm instrument stability and quantify pumped volume.

The equivalent spherical diameter (ESD, μm) was calculated by comparing forward scatter to silica beads (1.05–4.93 μm, Bangs Laboratories). Data were processed using FlowJo and R. Biovolume (BV, μm^3^) was derived as $\mathrm{BV}=\frac{4}{3}\cdot \pi \cdot {\mathrm{ESD}}^{3}$, assuming spherical cell morphology.

#### 2.4.2 Alkaline phosphatase activity

APA was monitored 3 h after each light–dark transition: at L3 and D3, and was quantified using the fluorogenic substrate 4‐Methylumbelliferone‐Phosphate (MUF‐P) (Hoppe 2003) at a final concentration of 125 μmol L^−1^. Samples (210 μL) were incubated in triplicate in black microplates at the culture temperature for 2 h. Control blanks were prepared from Milli‐Q water with added MUF‐P substrate. The fluorescence intensity was measured using a Perkin Elmer Victor3 Plate Reader fluorometer with an excitation wavelength of 365 nm and an emission wavelength of 450 nm. Fluorescence readings were done at t_0_ (time of MUF‐P addition), t_0_ + 30 min, t_0_ + 1 h, and t_0_ + 2 h. The increases in fluorescence, confirmed to be linear during the incubations, were transformed into enzyme activity rates using a standard curve obtained from a MUF solution with concentrations ranging from 0 to 100 μmol L^−1^.

#### 2.4.3 Particulate and dissolved nutrients

Samples for biochemical analyses were taken during the high‐frequency monitoring phase. The particulate organic matter was determined every 6 h, at L0, L6, D0, and D6. In each culture, two samples of 6.27 mL were collected independently, one to analyse particulate organic carbon (POC) and nitrogen (PON) by complete combustion and the other for particulate organic phosphorus (POP) quantification by wet oxidation. Culture samples were filtered on pre‐combusted (450°C—4 h) GF/C filters (nominal pore size 1.2 μm, Whatman). The filters were then dried and stored at 60°C before analysis. Filters for POC and PON were exposed to HCl fumes (4 h) before analysis using a CHNS elemental analyser (Thermo Fisher Scientific) calibrated with acetanilide (Lorrain et al. 2003). The lower detection limits of this elemental analyser were 0.41 μmol C and 0.07 μmol N on the filter. POP filters were oxidized before analysis on a segmented flow analyser (Skalar), according to (Pujo‐Pay and Raimbault 1994). The lower detection limits of the Skalar flow analyser were 0.01 μmol L^−1^ for P and 0.02 μmol L^−1^ for N. Cellular C, N, and P cell contents (fmol cell^−1^) were deduced using the precise volume of the sample and the cell abundance measured at each sampling time.

Nutrients were sampled at L0 and D0 in each culture, stored at −20°C, and later analysed. Samples of 30 mL were filtered using two GF/F filters combusted at 450°C for 4 h (nominal pore size < 0.5, Whatman). For quantifying dissolved inorganic nitrogen (DIN) and phosphorus (DIP), 7 mL of filtrate were analysed using a Segmented flow analyser (Skalar). Total dissolved nitrogen (TDN) and phosphorus (TDP) were measured using 10 mL of filtrate via the wet oxidation method of Pujo‐Pay and Raimbault (1994).

### 2.5 RNA Sampling and Preparation, Reverse Transcription, and Quantitative Real‐Time‐PCR

Every 3 h, 60 mL samples were collected and filtered through a 3 μm pore‐size polycarbonate membrane filter. The cells were resuspended in (0.2 μm filtered and autoclaved) seawater and centrifuged at 5000 rpm for 15 min. The pellet was transferred in 5 mL RNA later, immediately frozen in liquid nitrogen, and stored at −80°C until analysis. All measurements were carried out in duplicate. RNAs were extracted using the Qiagen RNA‐extraction kit following the manufacturer's instructions. An extra TURBO DNase (Invitrogen) digestion step was performed to eliminate the contaminating DNA. The RNA quality was assessed by a tape station system (Agilent). RNAs were quantified spectrophotometrically at 260 nm (NanoDrop 1000; Thermo Fisher Scientific). For cDNA synthesis, 200 ng total RNA and 0.5 μg Random Primers (Promega) were used with the GoScript Reverse Transcriptase (Promega) according to the manufacturer's instructions. Quantitative real‐time PCR (qPCR) analyses were performed on a CFX96 Real‐Time System (Bio‐Rad). The reaction volume was 15 μL and the final concentration of each primer was 0.5 μmol L^−1^. The qPCR cycling parameters were 98°C for 2 min, followed by 45 cycles of 98°C for 5 s, and 59°C for 10 s. The only exception was the 5′ND encoding gene, for which the melting temperature was 55°C. A final melting curve from 65°C to 95°C was added to determine the specificity of the amplification. To determine the amplification kinetics of each product, the fluorescence derived from the incorporation of SYBR Green Dye into the double‐stranded PCR products was measured at the end of each cycle using the SsoAdvanced Universal SYBR Green Supermix 2X Kit (Bio‐Rad, France). The results were analysed using Bio‐Rad CFX Maestro Software, version 1.1 (Bio‐Rad, France). The primers used in this study are listed in Table S1.

### 2.6 Statistics

For RT‐qPCR experiments (Figures 3, 4, and S2), the RNA 16S gene was used as a reference for normalization. The amplification efficiencies of each primer pair were 80 to 100%. All measurements were carried out in duplicate, a biological duplicate was performed for each point, and standard variations were calculated for each measure.

In Figures 5, 6, and Figures S3 and S4, we plotted individual replicate trends instead of averaged data with standard deviations. Potential outliers during the P_i_‐depleted and DOP recovery phases were identified using the Tukey fence test, based on the interquartile range (IQR). We flagged three data points: the cell abundance in replicate 2 at 4.75 d (4.41 × 10^6^ cells mL^−1^), replicate 1 at 13.0 d (3.44 × 10^6^ cells mL^−1^), and the POC cell content in replicate 1 at 1.0 d (163.21 fmol C cell^−1^). Outliers were defined as values below Q1‐ (1.5 × IQR), calculated from the full dataset for each phase. The first outlier (replicate 2, 4.75 d) was interpolated using adjacent time points (4.5 d and 5 d), the second (replicate 1, 13.0 d) was adjusted based on the overall trend, and the third (POC, 1.0 d) was replaced with the mean of the other replicates.

## 3 Results

### 3.1 Functional Genomics Survey of P_i_‐Related Genes in *Crocosphaera* Genomes

To evaluate the genetic capabilities of various *Crocosphaera* strains in adapting to phosphate (P_i_) deficiency, a comprehensive search for orthologs of proteins associated with transcriptional regulation in response to P_i_ deficiency, P_i_ import, and dissolved organic phosphorus (DOP) hydrolysis was conducted. To achieve this, protein functional domain analysis, supplemented with BLAST search, was performed for each category of proteins as described in the Experimental Procedures section. The findings for each functional category are outlined below and in Table S2.

#### 3.1.1 Signal Transduction and Gene Regulation

**FIGURE 1:**
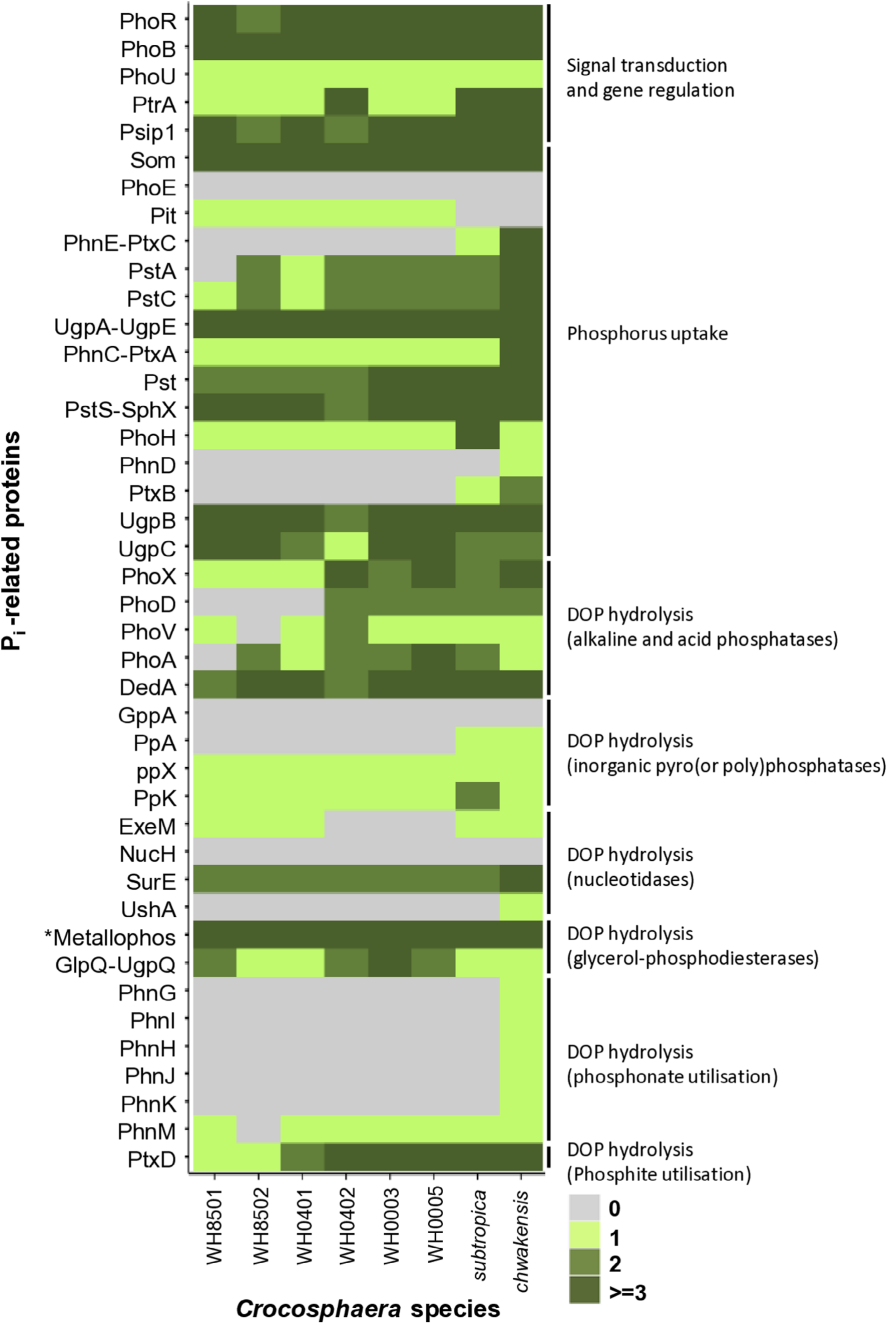
Heatmap representation of the number of P_i_‐related gene orthologs within the 8 *Crocosphaera* genomes. The colour scale and associated number from 0 to ≥ 3 represent the number of homologues found in each genome. Grey indicates that no ortholog sequence was found in the genome.

Genes encoding the PhoR histidine kinase sensor and PhoB response regulator were detected in all eight analysed *Crocosphaera* genomes, suggesting that the PhoBR system may primarily trigger the P_i_ limitation response (Figure 1, Table S2, Sheets 4,5). The PhoBR system activity varies with environmental phosphate levels. When P_i_ is abundant, PhoU dephosphorylates PhoB (Steed and Wanner 1993) and reduces both the activity and amount of the Pst transporter to limit P_i_ intake (Rice et al. 2009). This mechanism appears conserved across *Crocosphaera* genomes, as a *phoU* gene was detected in all of them (Figure 1).

In *Synechococcus* sp. WH8102, P_i_‐related gene regulation involves the CRP‐family transcriptional regulator PtrA, downstream of the PhoBR system (Ostrowski et al. 2010). Orthologs of *ptrA* were found in all *Crocosphaera* genomes, as were multiple copies of the *psip1* gene, a predicted CRP‐family member from *Prochlorococcus marinus* WH8102 (Figure 1, Table S2, Sheets 4,5). These findings suggest a role for CRP‐like regulators in P_i_ stress responses in *Crocosphaera*.

#### 3.1.2 Phosphorus Uptake

Gram‐negative bacteria, including *Crocosphaera*, can internalize phosphate (P_i_) through non‐specific porin channels in the outer membrane, which allow passive diffusion of small hydrophilic molecules. For instance, the major outer membrane protein Som in *Synechococcus* sp. PCC 7942 facilitates the uptake of such molecules (Umeda et al. 1996). Similarly, in *Crocosphaera*, P_i_ uptake may occur through the Som porin, as multiple putative Som‐encoding genes were detected across all *Crocosphaera* genomes (Figure 1, Table S2, Sheets 4,5). Unlike in *E. coli* where the PhoE porin, part of the Pho regulon, contributes to P_i_ uptake across the outer membrane (Korteland et al. 1982), no PhoE ortholog was identified in *Crocosphaera*.

To explore proteins facilitating P_i_ uptake across the inner membrane, we searched for orthologs of the high‐affinity Pst system and the low‐affinity Pit system. While Pit orthologs were found in *C. watsonii* , they were absent in *C. subtropica* and *C. chwakensis*.

The Pst system, an ATP‐binding cassette (ABC) transporter comprising PstC, PstA, and PstB subunits encoded by the *pstSCAB* gene operon of the Pho regulon, efficiently imports P_i_. PstS, a periplasmic P_i_‐binding protein, and SphX, a similar protein identified in *Synechococcus* PCC 7942, facilitate P_i_ delivery to PstC and PstA, with PstB driving ATP hydrolysis for P_i_ import into the cytoplasm (Rao and Torriani 1990; Aiba and Mizuno 1994).

**FIGURE 2:**
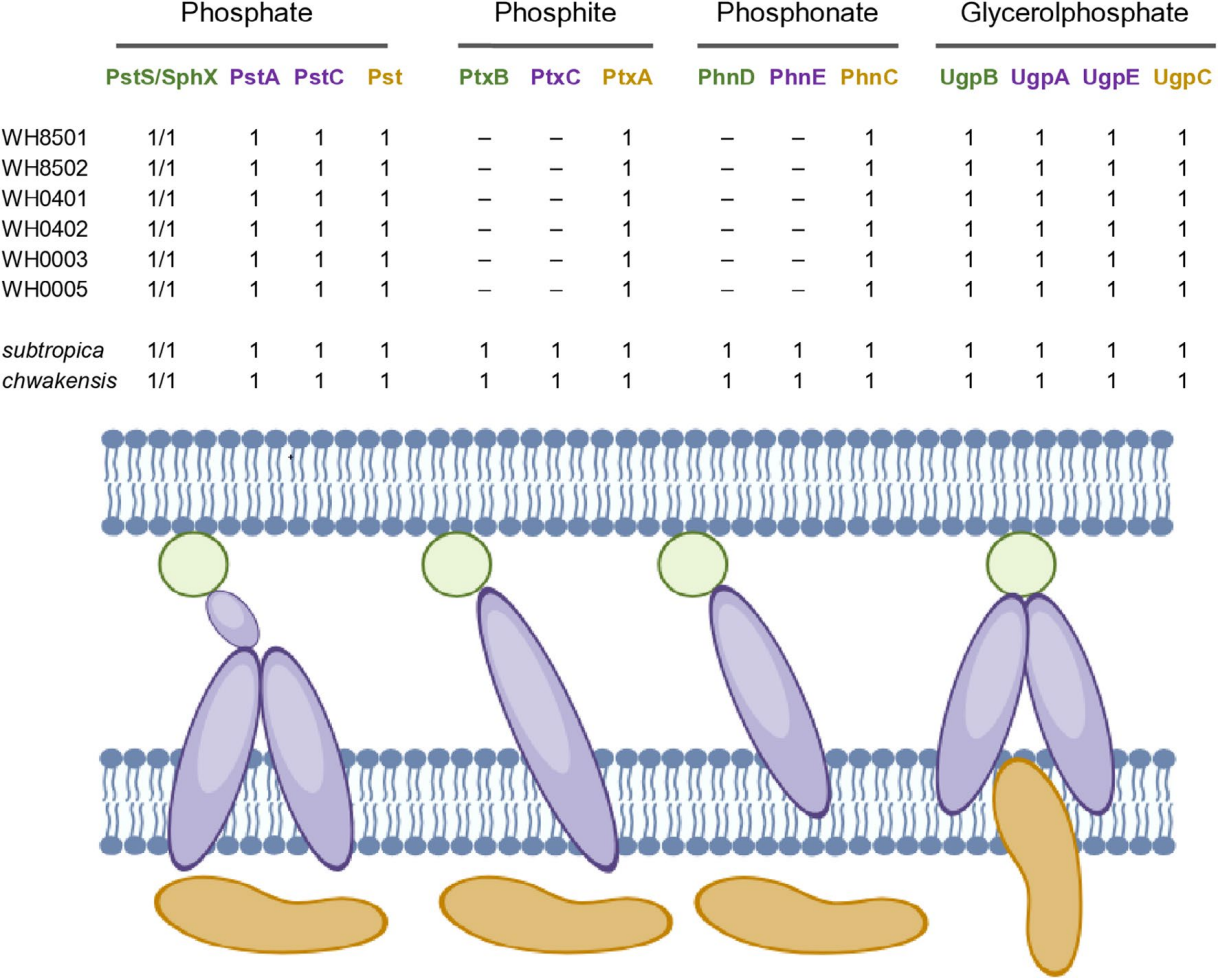
Schematic representation of phosphate (P_i_) transporter distribution across *Crocosphaera* strains. Label ‘1’ indicates the top‐scoring homologue (best hit) for each seed protein in every genome. (−) stands for the absence of ortholog. In each system, the P_i_ binding periplasmic protein is in green, the membrane subunit(s) in purple, and the cytoplasmic ATPase subunits in brown. The figure was drawn using BioRender.

In addition to P_i_, bacteria can use phosphites, phosphonates, and glycerol‐phosphate as phosphorus sources, internalized via ABC transporters similar to the Pst system. However, using functional domains to search for transporter subunit orthologs can result in overlapping identifications across different systems, as demonstrated in the heatmap (Figure 1). A comprehensive tBlastN analysis confirmed the presence of a complete gene set for the Pst system in all eight *Crocosphaera* strains (Figure 2, Table S2, Sheet 6). However, the *pstA* gene in *C. watsonii* WH8501 is a pseudogene, missing its corresponding protein in the Refseq database, likely due to sequencing errors.

Overall, our findings reveal that while all *Crocosphaera* strains likely internalize phosphoglycerol via the Ugp system, only *C. subtropica* and *C. chwakensis* possess complete gene sets for both the Ptx (PtxB, PtxC, PtxA) and Phn (PhnD, PhnE, PhnC) transporters (Figure 2, Table S2, Sheets 6). The presence of the *phoH* gene in all genomes, encoding a putative ATPase within the Pho regulon, suggests its potential role in adapting to P_i_ limitation (Figure 1).

#### 3.1.3 DOP Hydrolysis

We conducted a comprehensive analysis to identify genes that encode enzymes capable of liberating phosphate from various substrates, including phosphoesters (alkaline and acid phosphatases), phosphonates, polyphosphates (polyphosphatases), pyrophosphate (inorganic pyrophosphatase), and nucleotides (nucleotidases) (Table S2, Sheet 2). All *Crocosphaera* strains potentially possess the ability to hydrolyse dissolved organic phosphorus (DOP), as evidenced by at least one gene encoding a typical alkaline phosphatase in their genomes (Figure 1, Table S2, Sheet 5). Among the known bacterial alkaline phosphatases, only the *phoX* gene was universally identified across all *Crocosphaera* genomes. No *phoD* gene orthologs were detected in the genomes of *C. watsonii* WH8501, WH8502, and WH0401. Additionally, the *phoV* gene was absent in the *C. watsonii* WH8502 genome, and no *phoA* ortholog was identified in *C. watsonii* WH8501. Furthermore, all genomes harboured an ortholog of the *dedA* gene, which encodes an enzyme similar to alkaline phosphatase.

The utilization of inorganic pyrophosphate as a phosphate source appears confined to *C. subtropica* and *C. chwakensis* strains, as they exclusively contain the *ppA* gene encoding a pyrophosphatase. However, all strains seem capable of hydrolysing polyphosphates, with each genome containing at least one gene encoding PpX polyphosphatase. The presence of the *ppK* gene, potentially encoding a polyphosphate polymerase in all eight *Crocosphaera* strains, suggests a strategy for synthesizing polyphosphate reserves, potentially as an adaptation to either phosphate scarcity or irregular phosphate supply.

In terms of nucleotide hydrolysis, three nucleases—NucH, SurE, and UshA—were examined. A *surE* ortholog was found in all analysed genomes, a *ushA* ortholog only in *C. chwakensis*, and the *nucH* gene was absent in all eight genomes (Figure 1, Table S2, Sheet 5).

Our findings also indicate that *Crocosphaera* strains can utilize phosphoglycerol as a phosphate source, as all eight genomes contain at least one copy of a metallaphosphoesterase‐encoding gene, along with *glpQ* and *ugpQ*, which encode glycerol‐phosphodiesterases. The ability to use phosphonates seems limited to *C. chwakensis*, as the complete set of genes for hydrolysing these compounds (*phnGHIJMK*) and the transporter (*phnCDE*) was found only in its genome. Based on domain analysis, the PnhK protein might initially be misclassified as part of a transporter system due to its ATPase domain. However, functional analysis has confirmed that it belongs to the enzymatic complex responsible for phosphonate hydrolysis (Seweryn et al. 2015; Amstrup et al. 2023). Although only two *Crocosphaera* strains appear to possess the *ptxB* gene encoding a phosphite entry system, the gene responsible for phosphite utilization, *ptxD*, was identified in all eight genomes.

Gene conservation varied by morphotype, with small‐cell *C. watsonii* strains (WH0401, WH8501, WH8502) showing fewer orthologs (e.g., lacking *phoD*) compared to large‐cell *C. watsonii* (WH0003, WH0005, WH0402) and newly classified *Crocosphaera* spp. ( *C. subtropica* , *C. chwakensis*), which retained more alkaline phosphatase genes (*phoA*, *phoD*, *phoX*, *pafA/phoV*) (Table S2, Sheet 7).

### 3.2 Diel Expression of Genes Involved in P_i_‐Depleted and DOP‐Recovery Conditions

To investigate the genetic response of *C. watsonii* WH8501 to phosphorus‐depleted and DOP recovery conditions, we analysed the transcription of P_i_‐related genes every 3 h over 4 days under P_i_‐depletion and then over 2 more days after the same cultures were supplied with DOP.

**FIGURE 3:**
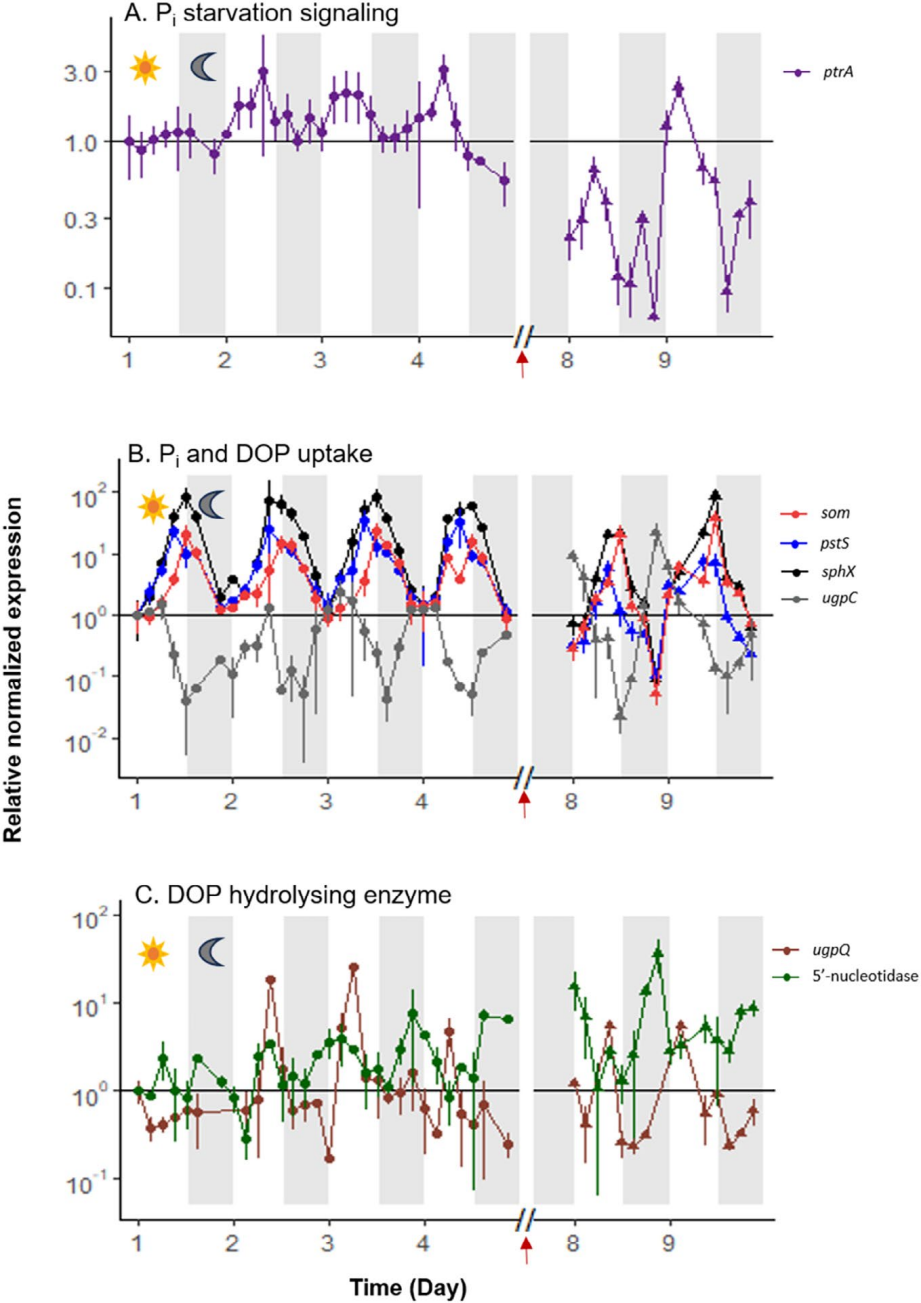
Relative normalized gene expression of *C. watsonii* WH8501 during the P_i_‐depleted phase (closed circles, days 1–4) and the DOP‐recovery phase (closed triangles, days 8–9). RT‐qPCR of the genes encoding for a regulator *ptrA* in purple (A), for a porin *som* in red, and transporters, *sphX* in black, *pstS* in blue, and *ugpC* in grey (B). Expression of the genes encoding for a glycerophosphoesterase *ugpQ* and a 5′‐nucleotidase are represented in brown and green, respectively (**C**). The Y‐axis scale is logarithmic. Time on the X axis is expressed in days, starting from the beginning of the high‐frequency monitoring phase, that is, five days after the transfer to a P_i_‐depleted medium. All points are normalized by the 16S expression at the same point and relative to the first sampling point (Day 1, L0). The horizontal line (y = 1) materializes the value of the first sampling point. The expression variability of biological and analytical duplicates is represented by error bars. White and grey shades represent light and dark periods, respectively and red arrows mark the time of DOP addition.

The expression of *ptrA* exhibited diel patterns with low overall amplitudes, as inductions measured during the light phases were approximately 2.5‐ to 3‐fold. Immediately after the addition of dissolved organic phosphorus (DOP), *ptrA* expression decreased by an average of 8‐fold during the two‐night phases following the shift, suggesting a possible role of this regulator in adaptation to changing phosphorus conditions (Figure 3A).

Gene expression patterns for P_i_ transporters like PstS and its homologue SphX showed synchronized diel rhythms, peaking at the light–dark transition. The *sphX* gene exhibited the highest fold change, averaging 100‐fold, while *som* and *pstS* transcripts showed similar patterns with about a 50‐fold change on average. These patterns persisted during the DOP‐recovery phase, indicating a sustained response in the cultures provided with DOP (Figure 3B). In contrast, *ugpC* expression varied significantly between the experimental phases, increasing by an average of 30‐fold during the first two dark phases following DOP addition. This highlights a distinct regulatory adaptation of the two transport systems (Pst and Ugp) to DOP availability, with the glycerol‐phosphate transport system being less expressed under P_i_ scarcity (Figure 3B).

In the category of genes involved in the P_i_‐stress response, the *ugpQ* gene, which encodes a glycerophosphoesterase‐like enzyme, exhibited the most pronounced cyclic expression patterns following the addition of DOP. Its expression, similar to that of *pstS* and *sphX*, peaked at the light:dark transition with an average 14‐fold increase (Figure 3C). The 5′‐nucleotidase gene showed oscillatory expression, primarily peaking in the dark phase, with a significant rise during the DOP‐recovery phase, increasing 50 to 100‐fold compared to a 5 to 10‐fold change during the depletion phase (Figure 3C). Other related enzyme‐encoding genes showed low and stable expression throughout the experiment with average fold changes ranging from 2 to 4 between the P_i_‐depleted and the DOP recovery phases (Figure S2). Overall, genes encoding enzymes capable of hydrolysing DOP were expressed from the depletion phase and continued post‐DOP supplementation.

### 3.3 Expression of Circadian Clock Genes

**FIGURE 4:**
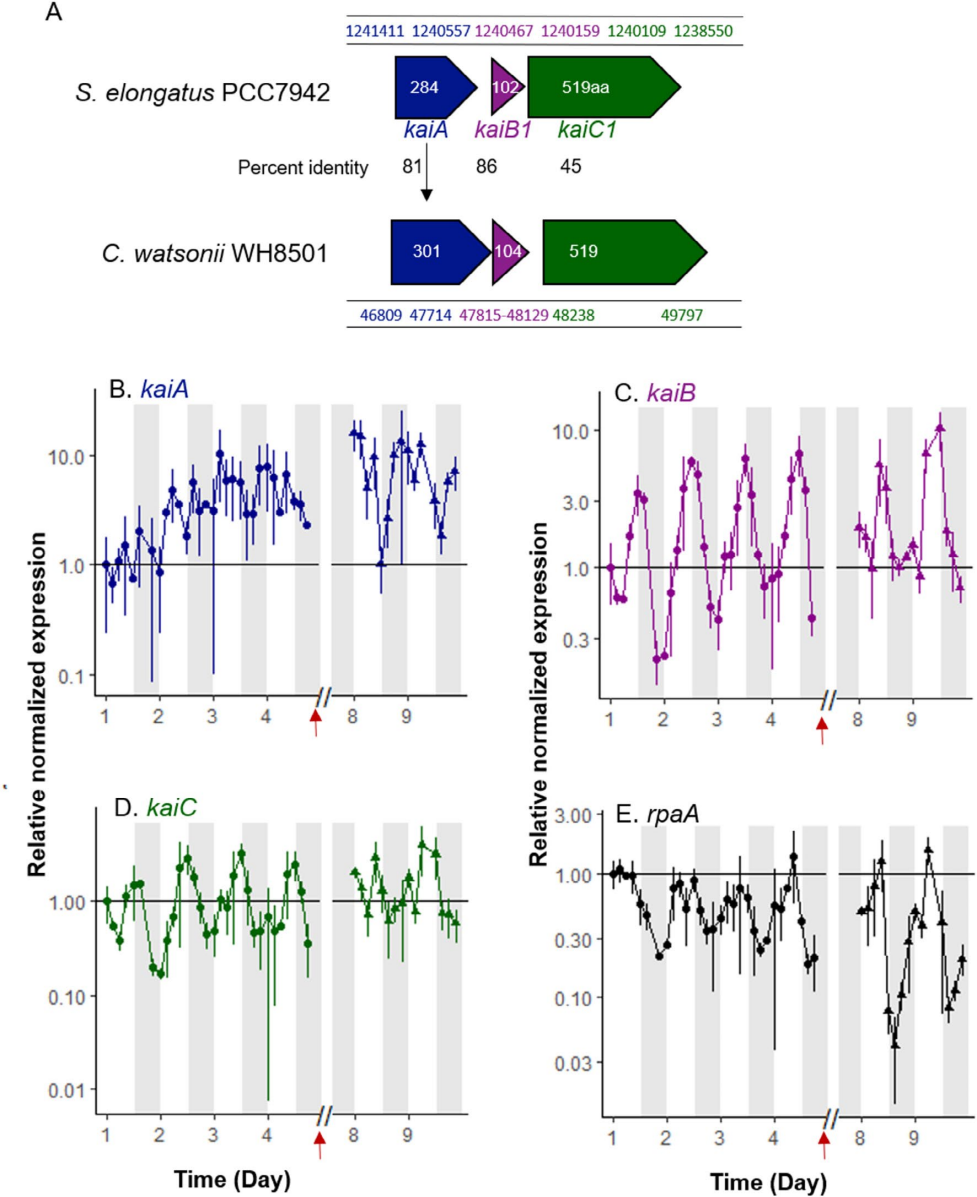
Gene cluster organization and genomic position of *Crocosphaera watsonii* WH8501 potential *kaiABC* and percent identity to *Synechococcus elongatus* PCC 7942 (A); relative normalized expression of *C. watsonii* potential circadian clock components *kaiA* (B), *kaiB* (C), *kaiC* (D) and regulator, *rpaA* (E) during the P_i_‐depleted phase (closed circles) and the DOP‐recovery phase (closed triangles). The Y‐axes are logarithmic. Time on the X axis is expressed in days, starting from the beginning of the high‐frequency monitoring phase, that is, five days after the transfer to a P_i_‐depleted medium. All points are normalized by the 16S gene expression at the same point and relative to the first sampling point (Day 1, L0). The horizontal line (y = 1) materializes the value of the first sampling point. Expression variability of biological duplicates and analytical duplicates are represented by error bars. White and grey shades represent light and dark periods, respectively, and red arrows mark the time of DOP addition.

The observed daily cyclic gene expression in our study likely results from circadian control by an internal clock. We investigated this by identifying orthologs of the *Synechococcus* clock genes *kaiA*, *kaiB*, and *kaiC*, along with *rpaA*, a transcriptional regulator gene, in the *C. watsonii* WH8501 genome. The *kaiA*, *kaiB*, *kaiC* genes were found clustered, with *kaiA* and *kaiB* likely forming an operon (Figure 4A). The *kaiB* and *kaiC* genes exhibited circadian expression with synchronous transcriptional peaks at the light–dark transition (and average fold changes of 10) (Figure 4C,D). The expression profiles of the kai genes in our study thus clearly differ from those observed by Kondo and Ishiura (2000) in *Synechococcus*. In contrast to *kaiB* and *kaiC*, the expression of the *kaiA* gene did not appear circadian and followed a different profile from that of *kaiB*, with variations ranging from 2 to 10‐fold over the analysed period (Figure 4B). Additionally, *rpaA* gene transcription peaked at night, aligning with its role in the clock's output system (Figure 4E). This supports the hypothesis that the internal clock may regulate the expression of genes involved in the P_i_‐stress response, which displayed diel cyclic expression.

### 3.4 Population Dynamics

We tracked cell abundance via flow cytometry during P starvation and DOP recovery to compare population dynamics and quantify growth rates, assessing how these conditions influenced exponential growth.

**FIGURE 5:**
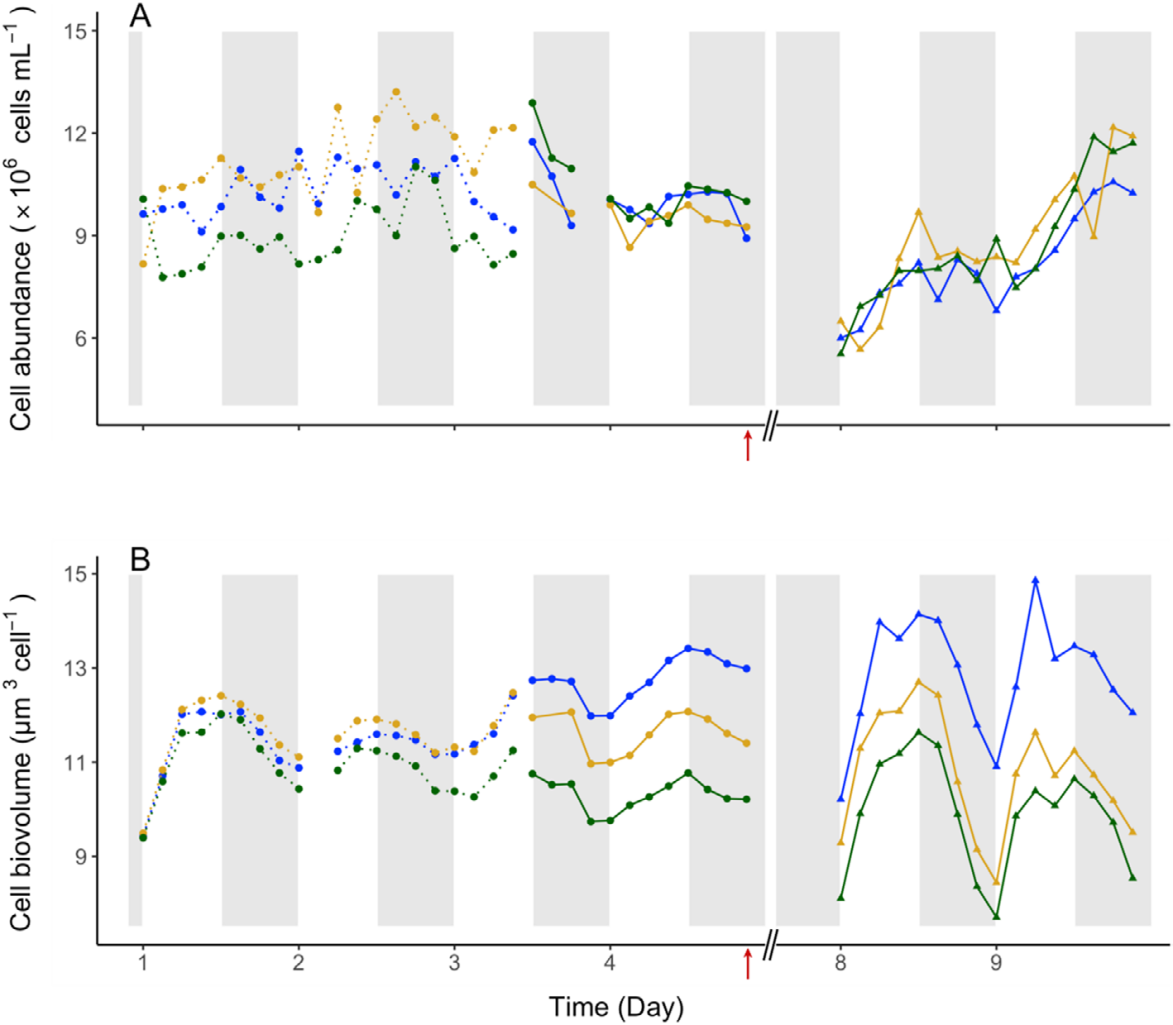
Diel fluctuations in *C. watsonii* cell abundance (top panel, ×10^6^ cells mL^−1^) and cell biovolume (bottom panel, μm^3^cell^−1^) represented for each culture replicate (blue, yellow, and green). Time on the X axis is expressed in days, starting from Day 1 at the beginning of the high‐frequency monitoring phase, that is, five days after the transfer to a P_i_‐depleted medium. The dotted lines represent sampling in the first culture triplicate and continuous lines represent samples taken in the second triplicate (see methods). The red arrows mark the time of DOP addition in the second triplicate; sampling of the DOP‐recovery phase was performed during Days 8 and 9. White and grey shades represent light and dark periods, respectively.

Upon transferring exponentially growing *C. watsonii* WH8501 cultures to a P_i_‐depleted medium, the initial cell concentration was 3.49 ± 0.22 × 10^6^ cells mL^−1^ (*n* = 6). A transient increase in cell abundance occurred in all cultures, with an average growth rate of 0.23 ± 0.03 d^−1^ (*n* = 6 replicates, 5‐time points) over the first four days (Days −4 to 0; Figure S3). This rate was slightly lower than that of the P_i_‐replete seed culture (0.26 d^−1^), likely due to higher cell densities and reduced irradiance. Nevertheless, the initial growth under P_i_ depletion followed exponential dynamics, with a maximum rate comparable to the P_i_‐replete culture, though the duration of the exponential phase was shorter. An inflection in the temporal fluctuation of the cell abundance was already visible from the 5th day of P_i_ depletion (Day 1). Cell abundance began to plateau, reaching 1.05 ± 0.09 × 10^7^ cells mL^−1^ (*n* = 6; Figures 5A, S3). Over the eight days of P_i_ depletion, cell numbers tripled (1.6 divisions on average), yielding an average growth rate of 0.14 d^−1^. Cell abundance initiated a decline on day 9 (Day 4 of P_i_ depletion) just as DOP was added to the remaining replicates. Within two days of DOP addition, growth resumed at μ_DOP_ = 0.31 ± 0.01 d^−1^ (*n* = 16 per replicate; average of three rates). Though this estimate is not very accurate as it is based on two days only (less than a full division cycle), the consistency across replicates and the clear shift in population dynamics confirm recovery and DOP utilization. The observed, notable difference between the maximum and average growth rates further highlights that the exponential phase was much shorter in duration than under optimal conditions and that the net growth was impaired under P_i_‐depleted conditions.

Under P_i_ starvation, cell size oscillated daily, increasing in the light and decreasing in the dark, with an amplitude of 0.13 ± 0.07 μm (*n* = 12; 4 time points per replicate). The average diameter was 2.79 ± 0.07 μm (*n* = 92), within the typical range for this strain (Webb et al. 2009; Dron et al. 2013; Rabouille et al. 2017). Biovolume averaged 11.41 ± 0.88 μm^3^ cell^−1^ (*n* = 92; Figure 5B, Days 1–4). After DOP addition, the amplitude of diel oscillations amplified (0.30 **±** 0.01 μm; *n* = 6), suggesting restored carbon fixation and consumption. The cell diameter averaged 2.77 **±** 0.14 μm (*n* = 48), and the biovolume averaged 11.19 ± 1.73 μm^3^ cell^−1^ (*n* = 48). In nutrient‐replete conditions, the average population cell size typically drops at mid‐light due to cell division (Dron et al. 2012, 2013). The absence of this drop during P_i_ depletion and its reappearance during DOP recovery further indicate disrupted division under P stress and its recovery upon DOP provision (Figure S4).

### 3.5 Dissolved Macronutrients and Alkaline Phosphatase Activity

We monitored dissolved N and P concentrations in *C. watsonii* WH8501 cultures and DOP hydrolytic activities to track the consumption of nutrients, which also inform on growth efficiency. Nitrate (NO_3_^−^) concentrations were close to zero (0.36 ± 0.28 μmol N L^−1^) during the whole experiment (Tables 1 and S4) since the medium did not contain any added nitrate. Organic (DON) and total (TDN) dissolved nitrogen levels were both lower during the P_i_‐depleted phase compared to the DOP‐recovery phase (9.19 ± 0.91 vs 19.10 ± 1.70 μmol N L^−1^ for DON and 9.44 ± 0.91 vs 19.68 ± 1.74 μmol L^−1^ for TDN; Tables 1 and S4). We hypothesize that N exudation processes were strongly reduced under P_i_ depletion and were expected to recover upon DOP addition. However, the measured DON at the end of the experiment was lower than the amount initially provided with the DOP, suggesting that cells had not yet fully recovered to resume N₂ fixation at a rate sufficient to generate excess N. Consequently, N exudation probably remained low during the recovery phase.

**TABLE 1:** Dissolved nutrient concentrations (μmol N or P L^−1^) and per‐cell alkaline phosphatase activities (APA, fmol cell^−1^ h^−1^) in replicates A, B, and C.

	Replicate	P_i_‐depleted	DOP recovery			
		(*n* = 8)	Day8	Day8	Day9	Day9
		D/L and L/D	D/L	L/D	D/L	L/D
Total dissolved phosphorus (TDP)	A	0.21 ± 0.03	3.33	1.71	1.53	0.98
	B	0.20 ± 0.01	5.01	3.84	3.00	1.33
	C	0.21 ± 0.02	3.63	2.10	1.86	0.60
Dissolved organic phosphorus (DOP)	A	0.20 ± 0.03	1.17	0.90	1.16	0.97
	B	0.17 ± 0.02	0.20	0.48	0.34	0.24
	C	0.17 ± 0.02	0.32	0.39	0.15	0.26
Phosphate (PO_4_^3−^)	A	0.01 ± 0.00	2.16	0.81	0.37	0.01
	B	0.03 ± 0.01	4.81	3.36	2.66	1.09
	C	0.05 ± 0.02	3.31	1.71	1.71	0.34
Total dissolved nitrogen (TDN)	A	9.62 ± 1.30	18.47	18.39	18.75	19.59
	B	9.36 ± 0.73	21.69	24.42	19.29	18.60
	C	9.35 ± 0.67	19.21	18.64	19.80	19.27
Dissolved organic nitrogen (DON)	A	9.36 ± 1.30	18.17	18.16	18.47	19.37
	B	9.12 ± 0.73	20.60	23.84	18.78	18.02
	C	9.09 ± 0.68	17.55	18.05	19.27	18.94
Nitrate (NO_3_^−^)	A	0.26 ± 0.05	0.30	0.23	0.28	0.22
	B	0.24 ± 0.04	1.09	0.58	0.51	0.58
	C	0.26 ± 0.06	1.66	0.59	0.53	0.33

		L3 and D3	L3	D3	L3	D3
APA	A	1.21 ± 0.73	2.56	4.59	3.43	3.28
	B	1.78 ± 0.76	5.47	6.06	5.02	5.83
	C	1.74 ± 0.84	5.43	7.17	5.64	4.38

*Note:* In the P_i_‐depleted phase, values varied little between dark–light (D/L) and light–dark (L/D) transitions, and so were averaged ± standard deviation in the culture over the entire monitored period. In the DOP‐recovery phase, macronutrient concentrations are reported for the dark–light (D/L) and light–dark (L/D) transitions of days 8 and 9, and the APA at L3 and D3.

During the P_i_‐depleted phase, the measured total dissolved P (TDP) in all cultures showed mean concentrations as low as 0.21 ± 0.02 μmol P L^−1^, with 0.03 ± 0.02 μmol P_i_ L^−1^ and 0.18 ± 0.03 μmol DOP L^−1^ (Table 1). As a corollary, the related DOP and P_i_ uptake rates were close to zero. Three days after the DOP addition (Days 8 and 9), DOP concentrations had dropped from 21.4 μmol P L^−1^ (upon DOP addition) to a range between 0.15 and 1.17 μmol P L,^−1^ while phosphate levels were between 2.16 and 4.81 μmol P L^−1^ on Day 8 and showed a decreasing trend over the two days monitored (Tables 1 and S4). Between the time of DOP addition (end of Day 4) and Day 8, the average TDP uptake rates were 1.59, 1.72, and 1.35 fmol P cell^−1^ d^−1^, respectively, in each replicate and decreased to 0.56, 0.29, and 0.45 fmol P cell^−1^ d^−1^ between Day 8 and Day 9 as DOP levels were already back down to the micromolar range and cell abundance had increased by a factor of 1.4 to 2.4. The magnitude of the average TDP uptake rate over the first four days of recovery is very congruent with the observed average P content of 2.6 fmol P per cell, given that the population does not divide every day. The lack of data on the days immediately following the DOP addition prevented us from quantifying possibly higher uptake rates that may have occurred at this time. These results strongly suggest that, whether extracellularly or internally (in the periplasm or the cytoplasm), most of the DOP added was rapidly hydrolyzed and the liberated P_i_ was mostly incorporated and conserved in cells. The APA assay was used to assess the production and activity of DOP‐hydrolyzing enzymes. The expression of alkaline phosphatases is part of the cell response to phosphate limitation in cyanobacteria (Ray et al. 1991; Scanlan et al. 2009; Tetu et al. 2009; Torcello‐Requena et al. 2024), including diazotrophic strains (Dyhrman and Haley 2006; Rabouille et al. 2022). The APA yielded rather low and stable activities during the P_i_‐depleted phase, with an overall average recorded activity of 1.56 ± 0.79 fmol cell^−1^ h^−1^ (*n* = 22). Rates increased 3.15‐fold during the DOP‐recovery phase, with an overall average of 4.90 ± 1.32 fmol cell^−1^ h^−1^ (*n* = 12) (Tables 1 and S4). To assess heterotrophic bacterial contributions to APA, we compared whole and pre‐filtered (< 3 μm) samples. Whole samples consistently showed higher APA (average + 35%), though proportions varied temporally. Flow cytometry revealed imperfect size separation, with some *C. watsonii* cells passing through the filter and some heterotrophic bacteria retained in larger fractions. Additionally, any extracellular enzymes from *C. watsonii* would remain in filtrates. While we could not quantitatively separate *C. watsonii* enzymatic contribution, the significantly higher APA in whole samples provides direct evidence that *Crocosphaera* itself produces hydrolytic enzymes, whether in attached extracellular form and/or within its periplasmic space.

### 3.6 Particulate Carbon, Nitrogen, Phosphorus, and C:N:P Ratios

The total carbon (C), nitrogen (N), and phosphorus (P) in the *C. watsonii* WH8501 biomass, along with their stoichiometry, were monitored and normalized by population abundance to determine cellular contents. We assume that all particulate C, N, and P are components of the cells. During prolonged experiments like a 9‐day phosphorus depletion, cell death can be expected. Bulk particulate C:N:P measurements include live cells and cellular debris from lysed apoptotic cells, the latter contributing to the dissolved organic pool. Therefore, the particulate fraction mainly represents viable cells but may include fragments from decaying cells.

**FIGURE 6:**
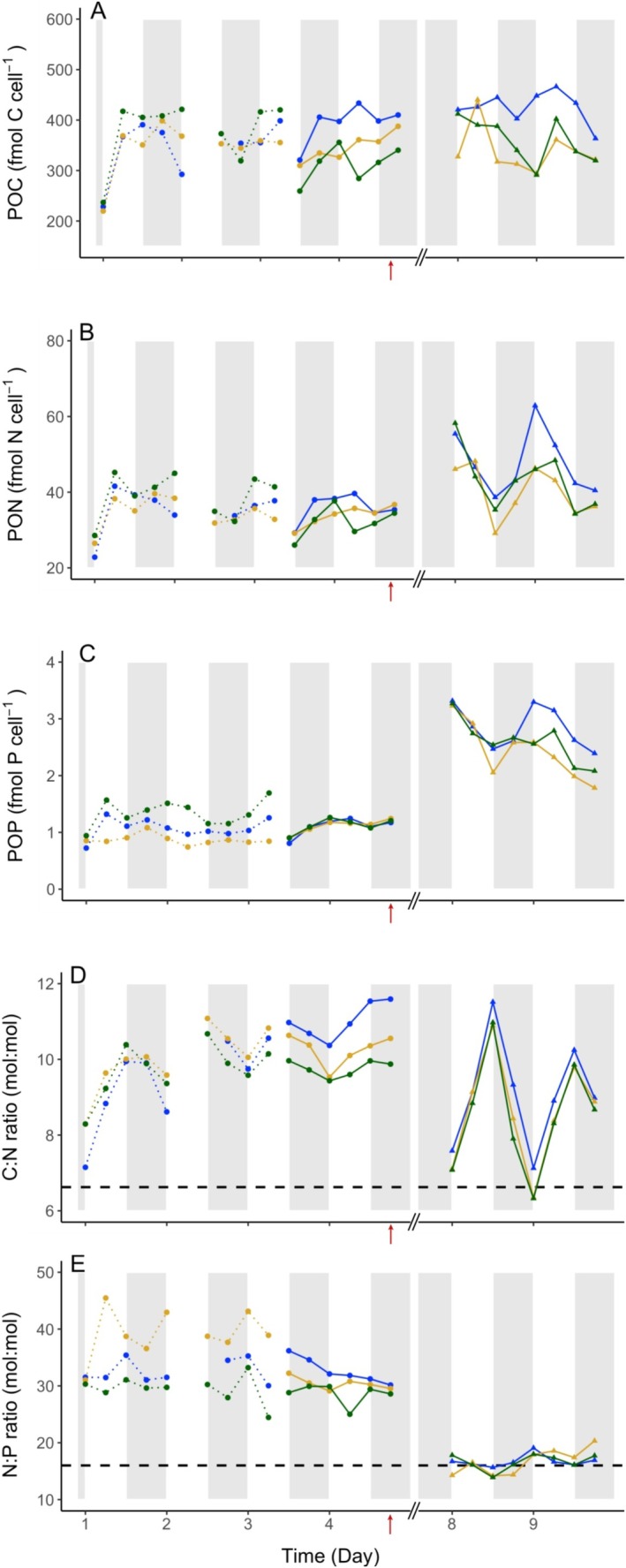
Diel fluctuations of *C. watsonii* C, N, and P cell contents in each replicate (blue, yellow, and green) during the P_i_‐depleted (Day 1 to Day 4) and DOP‐recovery (Day 8 and Day 9) phases. Particulate organic carbon (POC, fmol C cell^−1^, A) and particulate organic nitrogen (PON, fmol N cell^−1^, B) were used to estimate the C:N ratio (mol:Mol, D). Particulate organic nitrogen (PON, fmol N cell^−1^, B) and particulate organic phosphorus (POP, fmol P cell^−1^, C) were used to estimate the N:P ratio (E). Each content was normalized by the cell abundance estimated at the same time point. Time on the X axis is expressed in days, starting from Day 1 at the beginning of the high‐frequency monitoring phase, which was five days after the transfer to a P_i_‐depleted medium. The dotted lines represent sampling in the first culture triplicate and continuous lines represent samples taken in the second triplicate (see methods). The red arrows indicate the time of DOP addition. The dashed horizontal line represents the Redfield ratio. White and grey shades represent light and dark periods, respectively.

During the four days monitored in the P_i_‐depleted phase, the cellular carbon (POC) and nitrogen (PON) contents fluctuated somewhat irregularly, without a consistent diel pattern from day to day (Figure 6A,B,D). Although irregular, POC variations stayed within a relatively stable range between 228.3 and 433.6 fmol C cell^−1^, with an average content of 354.9 ± 52.8 fmol C cell^−1^ (*n* = 44) (Figure 6A). The PON also varied in a rather conserved range between 22.8 and 45.2 fmol N cell^−1^, with an average content of 35.6 ± 4.9 fmol N cell^−1^ (*N* = 44) (Figure 6B). A decreasing trend of 7.1 fmol C lost per cell and day, and 1.7 fmol N lost per cell per day were observed over the last 4.75 days of the P_i_‐depleted phase. Compared to the Redfield proportion, the decrease in N content was about 1.6 times faster than that of C content. Unlike the steady diel oscillations in the C and N contents observed in exponentially growing cultures, the more erratic changes in this data set suggest partial impairment of both C and N metabolism over these days. The resulting C:N ratio averaged 10 ± 0.7 (*n* = 44), which drifted far above the canonical Redfield ratio, reaching values between 10 and 12 on the last day of the P_i_‐depleted phase. The C:N stoichiometry was also much higher than that reported for unicellular diazotrophic cyanobacteria in other studies. For instance, Tuit et al. (2004) observed an average C:N ratio of 8.6 ± 1.8 for *Crocosphaera* spp. under a 14 h:10 h light:dark regime, while Mohr et al. (2010) and Dron et al. (2012) noted daily fluctuations of 6.5 to 8.5 and 4.96 to 8.76, respectively, in *C. watsonii* WH8501 cultures under a 12 h:12 h light:dark regime. The present ratios also exceed those observed in *Prochlorococcus* and *Synechococcus*, which range from 5 to 5.7 in nutrient‐rich conditions and 7.1 to 7.5 under phosphate limitation (Bertilsson et al. 2003). The significantly higher C:N ratio in the current experiment indicates a skewed N stoichiometry: N incorporation was proportionally more impaired than C fixation. Data also suggest that cells in one culture replicate tended to present higher C, N, and P contents than the other two (Figure 6A–C, blue curve). However, cells were also somewhat larger in that replicate (Figure 6B); thus, when normalized by biovolume, C and N content appeared more consistent across all three replicates (Figure S4).

The cellular P content fluctuated between 0.72 and 1.69 fmol P cell^−1^, with an average of 1.1 ± 0.22 fmol P cell^−1^. This trend suggests that the average P content in cells remained relatively constant over the four days monitored in the P_i_‐depleted phase (Figure 6C). The resulting mol:mol N:P ratio varied without a clear diel trend, between 17.0 and 28.5, averaging 22.9 ± 2.9 (*n* = 47) (Figure 6E). This average is notably above the Redfield reference, indicating particularly low cellular P content relative to N. Under severe phosphate depletion, the P stoichiometry dropped significantly, while N stoichiometry also decreased, but the carbon content was less affected.

During the recovery phase in the presence of dissolved organic phosphorus (DOP), the cellular carbon content fluctuated within a wider range (291.2–466.4 fmol C cell^−1^) than during the P_i_‐depleted phase, with an average content of 375.0 ± 54.1 fmol C cell^−1^ (*n* = 24), which was 4.6% higher than in the P_i_‐depleted phase (Figure 6A). The cellular N content showed wider and more regular oscillations, ranging from 29.1 to 62.9 fmol N cell^−1^ (Figure 6B), and a 23.6% increase in the average N content (43.7 ± 8.1 fmol N cell^−1^; *n* = 24). As a result, the C:N ratio in cells followed marked diel oscillations around an average value of 8.7 ± 1.41 (Figure 6D), which, while still above the Redfield value of 6.6, was 13% lower than the average observed in the P_i_‐depleted phase. These fluctuations indicate a clear increase in net nitrogen fixation efficiency compared to the P_i_‐depleted phase, suggesting that both gross carbon and nitrogen fixation increased during the DOP recovery phase. Likewise, the particulate P content showed notably higher levels in the recovery phase, with a 2.4‐fold increase compared to the depleted phase (Figure 6C). POP fluctuated in the range of 1.8–3.3 fmol P cell^−1^ with an average content of 2.6 ± 0.4 fmol P cell^−1^. These results corroborate the strong decrease in DOP concentrations measured in the culture and point to efficient hydrolysis of DOP and incorporation of the released P_i_ into cells. The corresponding N:P ratio thus dropped to values in the range of 13.9–20.3, with an average of 16.7 ± 1.6 (*n* = 24), very similar to the Redfield ratio, which is half that observed under P_i_‐depletion (Figure 6E). These values align with reported N:P ratios for *C. watsonii* WH8501 under nutrient‐replete conditions, which vary widely from < 5 to >30 (Knapp 2012; Liu et al. 2020), indicating substantial variability in cellular P content.

## 4 Discussion

The impact of nutrient limitation on nitrogen fixation in the open ocean is a widely studied topic, given the significance of diazotrophs in the input of new nitrogen in the system. The current literature largely highlights the role of P_i_ in the growth and nitrogen fixation rates of filamentous cyanobacteria, such as *Trichodesmium* (Spungin et al. 2014; Rouco et al. 2018; Frischkorn et al. 2019; Cerdan‐Garcia et al. 2022; Zhang et al. 2022). However, our understanding of how P_i_ influences the physiology of unicellular photosynthetic diazotrophs, especially for *Crocosphaera chwakensis* and *subtropica*, remains limited, as does our knowledge of how P_i_ availability might affect the growth of the *Crocosphaera* genus. To address these questions, we conducted a multidisciplinary study that included the functional analysis of eight *Crocosphaera* genomes from three different species ( *C. watsonii* , *C*. *chwakensis*, and *C. subtropica* ), together with an experimental monitoring of *C. watsonii* WH8501 exposed to a long P_i_ depletion.

We identified genes involved in P_i_ import, DOP utilization, perception of P_i_ limitation, and regulation of P_i_ stimulus expression in the eight analysed genomes, noting some variations in the composition of these genes (Figure 1, Table S2). Regarding import, all eight strains contained a complete high‐affinity P_i_ uptake system. Our analysis refined the identification of these genes, correcting ambiguities caused by annotation errors. For instance, prior studies identified two copies of the *pstA* gene in this genome; however, our research shows that what was previously annotated as two separate 100 bp fragments in the NCBI database (Shi et al. 2010) is a single *pstA* gene. Additionally, while the gene annotated as *phoA* in the *C. watsonii* WH8501 genome was thought to indicate the presence of alkaline phosphatase (Dyhrman and Haley 2006), our findings do not support the presence of a *phoA* gene. Instead, our analysis reveals that the gene in question encodes a 5′ nucleotidase. The only alkaline phosphatase encoding gene we found in the genome of this strain is *phoX*. Accurate identification of genes is crucial for guiding future functional investigations and advancing our understanding of biological processes, and the present study demonstrates how careful re‐examination using the same computational approach can correct initial misannotations, a well‐recognized challenge in genomic analysis.

We conclude that the eight *Crocosphaera* strains have a similar PstS ABC transporter (Figure 2). A gene encoding the low‐affinity P_i_ permease PitA was also identified in *C. watsonii*. In contrast, no *pitA* ortholog was found in the genomes of *C. subtropica* and *C. chwakensis* (Figure 1), which is surprising given their coastal habitats where phosphorus is presumably abundant. The absence of Pit suggests alternative P_i_ uptake mechanisms in these strains.

Genes encoding a complete phosphite and phosphonate transport system are absent in the genomes of *C. watsonii* strains, but are present in *C. subtropica* and *C*. *chwakensis*, indicating a possible adaptive speciation event (Figure 1, Figure 2, Table S2). This is puzzling as an inverse relationship was highlighted between phosphonate catabolic genes and P_i_ availability across a range of marine basins, suggesting that phosphonates are a source of phosphorus for the bacterioplankton in oligotrophic areas (Sosa et al. 2019; Lockwood et al. 2022). Future metatranscriptomic surveys will be necessary to determine whether the genes involved in phosphonate uptake and degradation are expressed and functionally active in *C. subtropica* and *C*. *chwakensis*. The basis for this specificity remains unclear. The most notable distinction of *C. chwakensis* is its occurrence in the Arctic Ocean (Shiozaki et al. 2023), unlike other *Crocosphaera* strains found exclusively at lower latitudes. This polar distribution suggests potential metabolic adaptations beyond cold tolerance, though little physiological or biogeographic data exist for this strain. In the Arctic, nitrate—not phosphate—is the primary limiting macronutrient (Tuerena et al. 2022). While phosphate concentrations there fall below micromolar levels, they remain higher than in oligotrophic oceans (< 10 nmol L^−1^; Whitney and Lomas 2019), implying phosphate competition is no more severe in polar regions. Thus, *C. chwakensis* Arctic distribution likely does not reflect a unique dependence on phosphonates to alleviate P_i_ stress. Further complicating this hypothesis, Acker et al. (2022) noted that microbial cells rarely combine phosphonate production and consumption capabilities. Their work with *Prochlorococcus* SB showed that phosphonate synthesis (constituting up to 40% of cellular P) did not serve as a P storage mechanism (Acker et al. 2022). Similar investigations in *C. chwakensis* could clarify whether its phosphonate use aligns with storage or alternative metabolic strategies.

In cultures, cell abundances can be orders of magnitude higher than in nature, and light gradients are also steeper. Consequently, the nutrient demand by the total population is also several orders of magnitude larger. We therefore stress the fact that direct comparisons of nutrient concentrations cannot be made between cultures and the natural environment. Reaching phosphate concentrations of 0.2 μmol L^−1^ with a population in the order of 10^7^ cells mL^−1^ represents a very severe nutrient depletion. Biochemical assays using the MUF‐P reagent have demonstrated DOP hydrolytic activities in cultures of *C. watsonii* grown with DOP supplementation (Pereira et al. 2019; Rabouille et al. 2022). Similarly, we observed comparable activities when P_i_‐depleted cultures were shifted to the DOP‐recovery phase (Tables 1 and S4). In their experiment, Pereira et al. (2019) observed an increase in APA in cultures during the 72 h following a transfer to a P_i_‐depleted medium, which confirms that APA responds to P_i_ stress within hours. In contrast, APA was low in our cultures, but our measurements were taken after 5 days of P_i_ starvation, that is, on a longer time scale. In parallel, the monitored C, N, P cellular composition points to a strongly skewed cell stoichiometry, with low N contents and very low, possibly minimal, P content (Figure 6). The synthesis of hydrolytic enzymes was therefore likely impaired by the lack of nutrients and cellular reserves. We suspect that the extended stress conditions had brought cultures in such a P‐depleted state that cells could no longer devote sufficient energy (ATP) and possibly also nitrogen and phosphorus to sustaining an active synthesis of hydrolytic enzymes. But as we added DOP, APA was soon restored. We conclude that the APA level that we measured at the end of the P_i_‐depleted phase, although much lower than that we measured three days after the addition of DOP, was not null. Some hydrolytic enzymes may also have remained active for a few days. This activity must have been sufficient for cells to start acquiring P again, which re‐launched both the P and N metabolism, as demonstrated by the wider amplitude of the cellular N and P contents (Figure 6), allowing cells to devote cellular C, N, and P to the synthesis of hydrolytic enzymes.

In the 3733 bacterial APase sequences gathered from the Global Ocean Sampling Expedition, Luo et al. (2009) found that 30% were extracellular, 17% cytoplasmic, 41% cytoplasmic, 12% outer membrane, and 0.9% inner membrane. The present work highlights that several enzymes could potentially be involved in DOP utilization in *Crocosphaera*. If not due to alkaline phosphatases, the DOPs hydrolytic activity might be attributed to enzymes such as nucleotidases or glycerol phosphatases, should these be able to cleave MUF‐P. The genes encoding these enzymes were transcribed in our experiments, but their sequences lack periplasmic targeting sequences (Table S3). Since the MUF‐P substrate cannot penetrate intact cell membranes and only reacts with periplasmic or extracellular enzymes (Luo et al. 2009), the measured alkaline phosphatase activity (APA) in healthy cells likely reflects PhoX activity; the only predicted extracellular phosphatase in our dataset capable of hydrolysing both MUF‐P and environmental DOP compounds. This conclusion is supported by PhoX extracytoplasmic localization prediction (Table S3). However, we must consider that prolonged P_i_ starvation (≥ 9 days) may have compromized membrane integrity in some cells. Under these stressful conditions, a subpopulation could have entered apoptosis or experienced lysis, potentially releasing cytoplasmic nucleotidases and glycerol phosphatases into the environment.

Recently, a novel alkaline phosphatase enzyme (called Psip), which shares low sequence similarity with the PhoA, D, V, and X enzymes, was identified in some *Prochlorococcus* and *Synechococcus* strains (Torcello‐Requena et al. 2024). This enzyme had initially been designated Psip1 (for Phosphate‐induced protein 1) (Ostrowski et al. 2010). We discovered an ortholog of *psip1* exclusively in the genome of *C. chwakensis*. Therefore, the related enzyme cannot be responsible for the DOP hydrolytic activity observed in our experiment on *C. watsonii* WH8501. Note that the name Psip is also used in the literature for a transcriptional regulator involved in P_i_‐stress signalling in the annotation of *C. watsonii* genome (Figure 1). The multiple uses of the same name for an enzyme and a regulator, as well as annotation errors (as pointed out above for *phoA*), are the source of biases and confusion in the current databases. This underscores the need for improved annotation of P_i_‐related genes in cyanobacterial genomes.

The P_i_ stress also resulted in changes in physiological parameters, such as cell size and elemental C, N, P contents (Figures 5, 6). The survival of the cultures throughout the long P_i_ starvation period, as indicated by the relatively stable cell abundance and higher average C:N and N:P ratios relative to the Redfield ratio, reflects a certain metabolic resilience facing P_i_ depletion (Figure 6, Tables 1 and S4). The dampened increases and decreases in cell volume, C, N, P contents, and skewed C:N and N:P ratios observed at the end of the P_i_‐depleted phase altogether indicate that under phosphorus starvation, cells managed to maintain a basal activity but showed a relative inability to divide. If some cell division occurred, it could at most compensate for cell decay (Figure 5, Tables 1 and S4). All the measured parameters (cell abundance, cell contents, particulate, and dissolved fractions) indicate that cells maintained their viability during the limitation period. The restored cells C, N, P contents and their diel cyclicity following the addition of DOP‐containing medium reinforce this conclusion, demonstrating a recovery of growth supported by the DOP addition (Figures 5, 6). In *C. watsonii* cultures, the genes linked to phosphate uptake (*som, sphX, pstS, ugpC*), signalling (*ptrA*), and DOP utilization (5′ nucleotidase, *ugpQ*) are transcriptionally induced under phosphate limitation and further responsive to DOP supplementation. This induction pattern, as observed in Figures 3 and S2, suggests their role in mediating the cellular response to this environmental stress.

What could not be asserted in the present work is whether there is a strong diel periodicity in DOP acquisition. While P_i_‐stress response genes exhibit diel expression patterns (Figures 2, 3, 4, and S2), it remains unclear whether this translates to rhythmic protein production. The timing depends on mRNA stability, translation rates, and enzyme half‐lives. Some enzymes may persist beyond their synthesis day, potentially maintaining constant activity despite cyclic gene expression.

Figure 6 suggests that the P content in cells increases more in the dark phase than in the light phase, but we would need more than a few light cycles to confirm this statement. If this is true, this could be consequent to a higher gene expression in the early dark than in the early light phases, and/or to the fact that the synthesis of DOP hydrolytic enzymes requires nitrogen, which is primarily acquired in the dark. Nonetheless, our findings reveal the metabolic resilience of *C. watsonii* and underscore the profound impact of P_i_ stress on both energetic processes and nitrogen metabolism. Additionally, they illustrate the crucial role of P_i_ stress‐response genes in this adaptive strategy.

At Station Aloha, the average concentrations of DOP and SRP measured over a year reveal that DOP concentrations are 5.6 to 8.5 times greater than those of SRP in the upper 75 m, and 0.9 to 4.7 times higher between 75 and 175 m in depth (Björkman and Karl 2003). In the marine environment, more than 80% of DOP is in the form of phosphoesters (Young and Ingall 2010; Bell et al. 2020), which *Crocosphaera* spp. can use. The phosphoesters:SRP ratio at Station Aloha would thus be in the range of 4.5–6.8 in the upper 75 m and 0.7–3.8 between 75 and 175 m. So, while SRP concentrations are below 50 nmol L^−1^ in the upper 75 m, the total, potentially available phosphorus concentrations may be close to 250 nmol L^−1^. Yet, forecasting to what extent the DOP pool may support *Crocosphaera* growth would require knowing the renewal rates of available DOP. Also, *Crocosphaera* must compete with other microorganisms for access to this resource, so deriving uptake rates for *Crocosphaera* alone would overestimate its actual utilization of the DOP resource. By monitoring ATP uptake rates, Björkman and Karl (2003) demonstrated that the DOP and SRP pools support the P demand of the microbial community equivalently at Station Aloha, and that the utilization of the DOP fraction increases as SRP availability decreases. A refined analysis with an estimation of the different size classes to ATP uptake led Björkman et al. (2018) to conclude that most of the P derived from ATP is incorporated in the < 2 μm size fraction at Station Aloha, except for the P_i_‐depleted sampled stations and those sampled during a bloom, for which the > 2 μm size fraction showed much higher ATP uptake rates. Our laboratory‐based results strongly support these observations, showing that the N_2_‐fixing cyanobacteria *Crocosphaera* spp. can switch to using DOP under P_i_‐depleted conditions. Additionally, our study emphasizes the importance of conducting more extensive temporal, in situ monitoring of the DOP pool and its components in the marine environment, alongside tracking production and consumption rates.

## Author Contributions

Conceptualization: Sophie Rabouille, Amel Latifi. Investigation: Emmanuel Talla, Chloé Caille, Sophie Rabouille, Amel Latifi, Olivier Crispi, Barbara Marie, Eva Ortega‐Retuerta, Yann Denis, Mireille Pujo‐Pay. Formal analysis: Emmanuel Talla, Amel Latifi, Sophie Rabouille, Chloé Caille, Vladimir Daric, Yann Denis. Validation: Amel Latifi, Emmanuel Talla, Sophie Rabouille, Yann Denis. Visualization: Chloé Caille, Emmanuel Talla, Amel Latifi, Sophie Rabouille. Supervision: Sophie Rabouille, Amel Latifi, Eva Ortega‐Retuerta. Paper writing: Amel Latifi, Sophie Rabouille. Funding acquisition: Sophie Rabouille.

## Conflicts of Interest

The authors declare no conflicts of interest.

## Supporting information


**Data S1.** Supporting Information.

**Table S2.** (Excell file).

**Table S4.** Source data for results presented in Table 1.

## Data Availability

The data that supports the findings of this study are available in the Supporting Information of this article.
