# Supplementary material for: Unveiling Crocosphaera Responses to Phosphorus Depletion: Insights From Genome Analysis and Functional Characterization
Source: Environ Microbiol. 2025 Jul 14;27(7):e70153. doi: 10.1111/1462-2920.70153 (PMC12260270; doi:10.1111/1462-2920.70153)
Supplement: Supplementary file 1 — Data S1. Supporting Information. [file EMI-27-e70153-s003.docx]

**Appendix**

**Appendix Supplementary Tables**

**Appendix Table S1.** Primer sequences and amplification conditions for the genes used in RT-qPCR

| **Gene** | **Primer** | **Sequence (5’-3’)** |
| --- | --- | --- |
| 16S    5’ND    *dedA*    Metallophophoesterase    *pstS*    *ptrA*    *som*    *sphX*    *surE*    *ugpC*    *ugpQ* | F  R  F  R  F  R  F  R  F  R  F  R  F  R  F  R  F  R  F  R  F  R | AAAGCTTACCAAGGCGACGA  GAAAATTCCCCACTGCTGCC   CCCTTGTAGAAACCCCACCT   TGTCACCAATTCTCCGGCTA  GCCCCTCCCAAGTTACACAA  TTTGGGCGTTTTGTCACCTT  ACAAATAGAAGCGATGTGGGC  AGAACCAGAAGCGTCGTGAT  CCGTAGTTCACCGTTCCGAT  AAGCGGTTACCCCTTCGTTT  TTTGTTAGGTTGGGCGCAAC  ATTCGACGGGCTAACTGACT  CGTGTGGCTAGTGATCCCTT  CTCCATTAGCCGGGTTAGCA  CGATAATGGGAATGGTGGGGT  AGGATTTTCTTGGGCCCGTT  CTTCATCAGCCCATTCGTGC  GGTGGGGTTTCTACAAGGGT   CTGGGTGTGGCAAAAGTACC   ACAGCAGGTTGTCTCACGAT  GCCTCTTATAATTGCTCACCGTG  TCAGCTACATTTGTCGTTTCTGA |
| *phoX* | F  R | ACAAAACAAAGGGGCTGCAA  AACACACACCAGCAAACTGG |
| *rpaA* | F  R | ATGTTGCCCAAAGTAGAC  GTTTGACCGTGACGCT |
| *kaiA*  *kaiB*  *kaiC* | F  R  F  R  F  R | GGGCGTAGTTGTTGTCTTGC  CGTCCATCAGTTCCATGTGC  CAAACTCTACGTCGCTGGTAAT  CCTCATAGAGAAGATCCAAACCA  GATGCAGCTCTGTGGTGAG  GAAGAACTGCGCGCATTA |

**Appendix Table S2 (Excell file):**

**Sheet 1. List of *Crocosphaera* genomes used in this study.** The cell size features were obtained from the literature.

**Sheet 2. Protein seeds (or reference proteins) used in this study.** Accession numbers (from Uniprot www.uniprot.org and Refseq www.ncbi.nlm.nih.gov databases) are indicated as well as the targeted molecules and the functional domain patterns (see **Experimental procedures**). The targeted molecule associated with each seed was obtained from the literature. Within the domain patterns, domains are shown in the order of appearance in the protein and separated with a star (*). Domain abbreviations and their functional descriptions of the domains are shown in **Sheet 3**. Other data were retrieved from the Uniprot or NCBI databases. Similar or identical seeds (from distinct organisms) are grouped into one seed (*e.g.* PhnC-PtxA).

**Sheet 3. List of functional seed domains**

**Sheet 4. List of transporter protein orthologs.**

**Sheet 5. List of protein orthologs.**

**Sheet 6. Distribution of best-hit transport protein orthologs in *Crocosphaera* species**

**Sheet 7. Distribution of protein homologs in the group of *Crocosphaera* species**

**Appendix Table S3: Prediction of cellular location of DOP-hydrolyzing enzymes in *C. watsonii* WH8501**

The presence of signal peptides Sec or Tat was analyzed using Signal IP-6.01 software (https://services.healthtech.dtu.dk/services/SignalP-6.0/)

**Appendix Table S4: Source data for results presented in Table 1**

**Appendix Supplementary Figures**

**
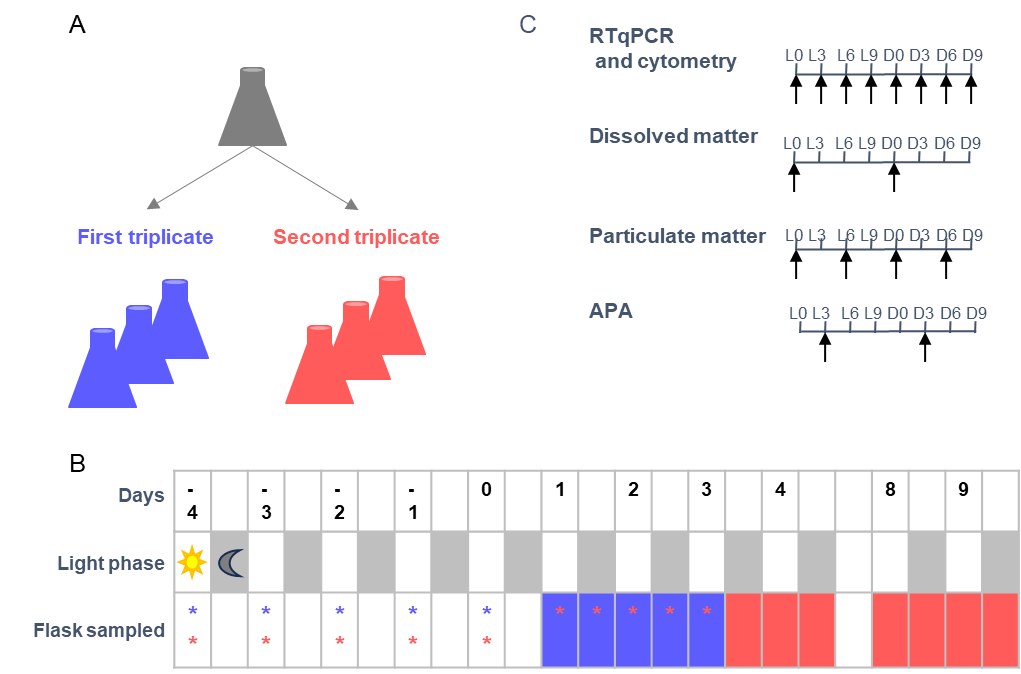
**

**Appendix Figure S1:** **A)** Biomass cultures were maintained in six flasks, with two biological replicates (indicated in blue and pink).

**B)** On Day –4, cultures were transferred to phosphorus-free (Pi-free) conditions.

Light phases are depicted by a sun (day) and a moon with gray bands (night).

Low-intensity sampling phase (Days –4 to 0): Daily cell abundance measurements (represented by a **star, ***) were taken.

High-intensity sampling phase (Days 1 to 9): Triplicate samples were collected, with blue and pink denoting the first and second triplicates, respectively. Sampling events are indicated by arrows.

**C)** Dark-light transitions (L0) occurred every 3 hours at L3, L6, L9, D0, D3, D6, D9, where L = light and D = dark.

**
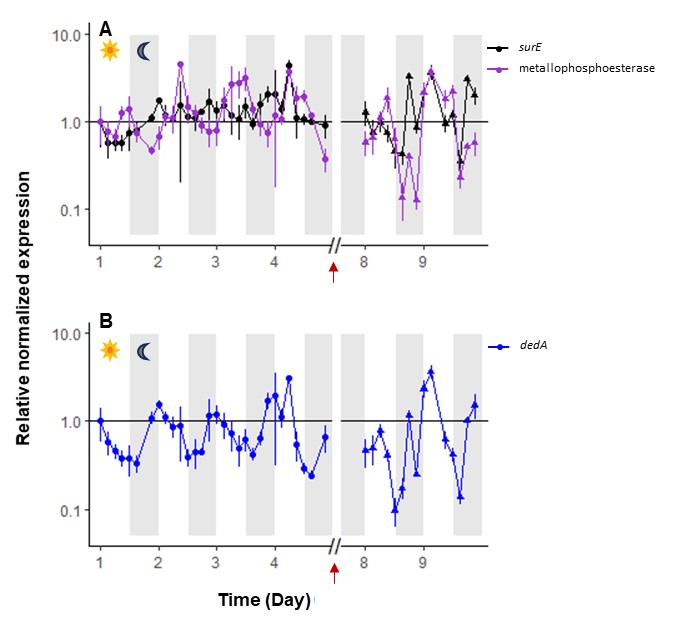
**

**Appendix Figure S2:** Relative normalized gene expression of *C. watsonii* during the P_i_-depleted phase (closed circles, left panels) and the DOP-recovery phase (closed triangles, right panels). RT-qPCR of the genes encoding a potential 5’-nucleotidase, *surE* (in black, **A**), a metallophosphoesterase (in purple), and the alkaline phosphatase-like, *dedA* (in blue, **B**). The Y-axis is logarithmic. All points are normalized by the 16S expression at the same point and relative to the first sampling point (Day 1, L0). Expression variability of biological duplicates and analytical duplicates are represented by error bars. White and grey shades represent the light and dark periods, respectively; red arrows indicate the day of DOP addition.


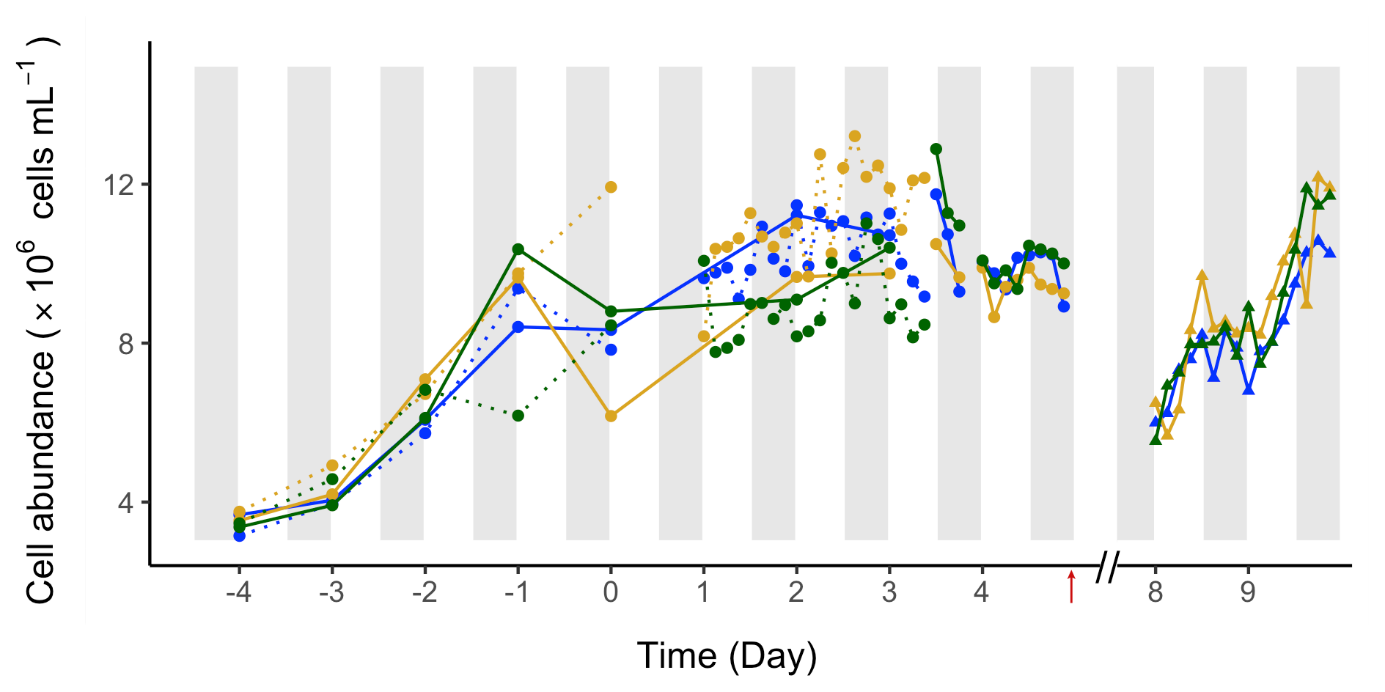


**Appendix Figure S3:** Diel fluctuations in *C. watsonii* total population cell abundance (× 10^6^ cell mL^-1^) measured in each triplicate (blue, yellow and green) over the entire P_i_-depleted period. Cultures were transferred to the P_i_-depleted medium on Day -4 and the high-frequency monitoring started on Day 1. The dotted lines represented sampling in the first triplicate and solid lines samples taken in the second triplicate. The red arrow marks the day of DOP addition and samples of the DOP-recovery phase were realized Days 8 and 9.
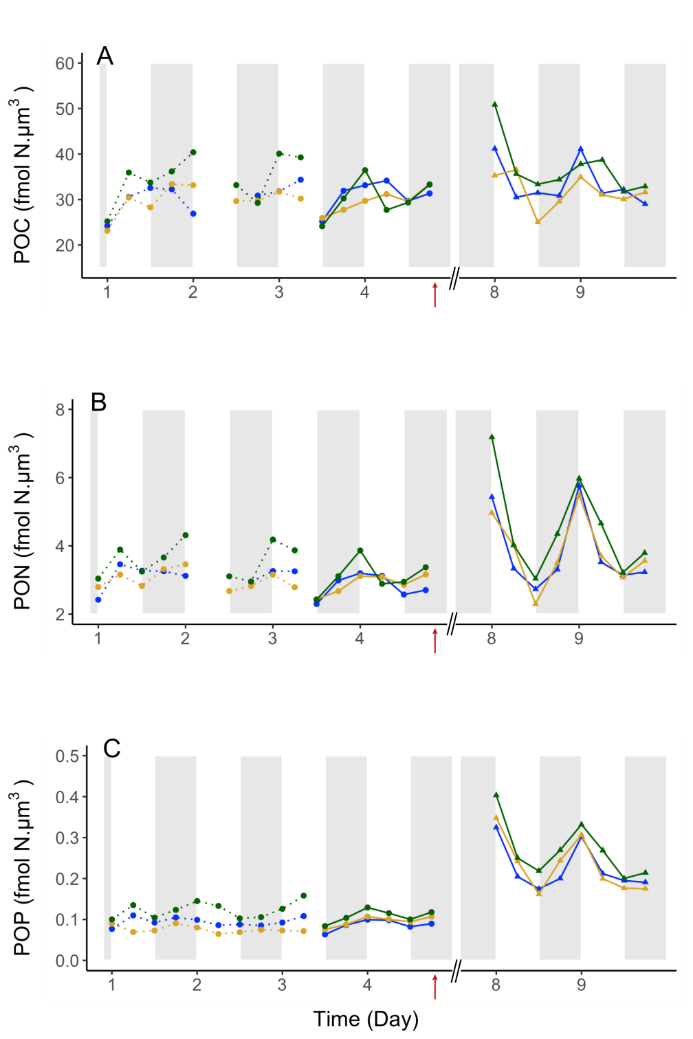


**Appendix Figure S4:** Diel fluctuations of *C. watsonii* C, N and P cell contents in each replicate (blue, yellow and green) during the P_i_-depleted (Day 1 to Day 4) and DOP-recovery (Day 8 and Day 9) phases. Particulate organic carbon (POC, fmol C µm^3^, A). Particulate organic nitrogen (PON, fmol N µm^3^, B). Particulate organic phosphorus (POP, fmol P µm^3^, C). Each content was normalized by the biovolume (µm^3^) estimated at the same time point. Time on the X axis is expressed in days, starting from the beginning of the high-frequency monitoring phase, five days after the transfer to a P_i_-depleted medium. The dotted lines represent sampling in the first culture triplicate, and continuous lines represent samples taken in the second triplicate (see methods). The red arrow indicates the time of DOP addition. The dashed horizontal line represents the Redfield ratio. White and grey shades represent light and dark periods, respectively.
